# Comparative Analyses of Targeted Myeloid Cancer Next-Generation Sequencing Panel in Fresh Blood, Bone Marrow and FFPE Material

**DOI:** 10.3390/ijms25063534

**Published:** 2024-03-21

**Authors:** Andrea Daniela Hobeck, Sophia Wendt, Saskia Krohn, Gudrun Knuebel, Stephan Bartels, Elisa Schipper, Christian Junghanss, Hugo Murua Escobar

**Affiliations:** 1 Clinic for Hematology, Oncology and Palliative Care, Medical Clinic III, Department of Internal Medicine, Rostock University Medical Center, Ernst-Heydemann-Strasse 6, 18057 Rostock, Germany; andrea_hobeck@web.de (A.D.H.); sophia.wendt@med.uni-rostock.de (S.W.); saskia.krohn@med.uni-rostock.de (S.K.); gudrun.knuebel@med.uni-rostock.de (G.K.); christian.junghanss@med.uni-rostock.de (C.J.); 2 Institute of Pathology, Department of Molecular Pathology, Hannover Medical School, Carl-Neuberg-Str. 1, 30625 Hannover, Germany

**Keywords:** next-generation sequencing, Oncomine^TM^ Myeloid Panel, amplicon sequencing performance, FFPE material, fresh blood/bone marrow material

## Abstract

Next-generation sequencing is a vital tool for personalized diagnostics and therapies in cancer. Despite numerous advantages, the method depends on multiple parameters regarding the sample material, e.g., sample fixation. A panel’s ability to ensure balanced pre-amplification of the regions of interest is challenging, especially in targeted sequencing approaches, but of significant importance to its applicability across hematological malignancies and solid tumors. This study comparatively evaluated the technical performance of the commercially available Oncomine^TM^ Myeloid Panel in fresh and Formalin-fixed paraffin-embedded (FFPE) material by using an Ion Torrent™ Personal Genome Machine™ System and Ion GeneStudio S5 System platform. In total, 114 samples were analyzed, including 55 fresh materials and 59 FFPE samples. Samples were sequenced with a minimum of one million reads. Amplicons with coverage below 400 reads were classified as underperforming. In fresh material, 49/526 amplicons were identified as performing insufficiently, corresponding with 18 genes. Using FFPE material, 103/526 amplicons underperformed. Independent of input material, regions in 27 genes, including *ASXL1*, *BCOR* and *BRAF*, did not match quality parameters. Subsequently, exemplary mutations were extracted from the Catalogue of Somatic Mutations in Cancer database. This technical evaluation of the Oncomine^TM^ Myeloid Panel identified amplicons that do not achieve adequate coverage levels and which need to be considered when interpreting sequencing.

## 1. Introduction

The understanding of cancer as a disease that is mainly based on genetic mutations enables modern diagnostics. In this context, next-generation sequencing (NGS) allows for the detection of genetic aberrations, such as somatic mutations, copy number variations, resistance mechanisms against chemotherapy, quantification of mutational burden and germline mutations, via a single method. 

By using targeted NGS, common mutations for specific cancer types can be determined. One striking example is myelodysplastic syndromes (MDS), a heterogeneous group of hematopoietic diseases with clinically unspecific phenotypes and with overlaps with other hematopoietic diseases. Here, oncogenic mutations aiding the definition of a diagnosis can be identified in up to 90% of patients using targeted NGS panels [1,2]. Another complex myeloid neoplasm is acute myeloid leukemia (AML), which has high mortality rates when untreated and is characterized by multiple somatic driver mutations. Rapid diagnosis and the initiation of adequate therapy are essential for a patient’s outcome. These diseases highlight how molecular genetic screening is indispensable in diagnostics and can be used for detailed disease classification [3,4]. Based on targeted NGS studies, amongst others, the World Health Organization (WHO) was able to classify the heterogeneous molecular landscape of AML [5] and MDS [6]. 

Compared with conventional methods like cytomorphology, targeted NGS enables the determination of a patient’s specific mutation profile. Results further allow individual risk stratification, especially in challenging cases [7]. In 2022, the International Consensus Classification (ICC) released a more genetically defined classification for AML and identified specific disease categories which may impact prognosis and therefore patient outcome [4]. For instance, specific mutations in *ASXL1* or *RUNX1* are associated with an adverse prognosis [3]. 

While targeted NGS enables precise, personalized cancer therapy, this is made much more complex and challenging due to interindividual heterogeneity. Nevertheless, NGS results have been found to reduce the number of allogeneic hematopoietic cell transplantations in AML patients by 20–25%, with the same overall survival rates [8]. Furthermore, NGS databases have the potential to identify individual drug targets or determine patients not benefiting from current therapy. This demonstrates that genetic features have become increasingly important in cancer care, in addition to clinical criteria. 

Despite the technological advances since its introduction, some challenges remain in NGS diagnostics. The NGS workflow consists of multiple steps, all of which having the potential to introduce errors. Therefore, quality control in every step, from library preparation to the sequencing process to data analysis, is essential for suitable and consistent sequencing performance. Automated workflows can optimize the sequencing process by minimizing handling mistakes, but the sequencing quality also depends on the characteristics of the region of interest itself. For example, regions with homopolymer sequences are associated with poor sequencing performance when using the Ion Torrent NGS systems [9]. 

Furthermore, the quality of input material significantly influences the overall sequencing performance, as nucleic acid yield and quality affect the sequencing performance independent of the sequencing platform [10,11,12]. Here, it should be first noted that, in general, bone marrow and peripheral blood are equally adequate sample sources for targeted NGS diagnostics [13,14]. For example, an AML diagnosis can be established from bone marrow and peripheral blood samples according to the 2022 recommendations of the European LeukemiaNet [3]. Formalin-fixed paraffin-embedded (FFPE) tissue samples are especially challenging input materials. As chemical preparation with formalin induces crosslinks between intracellular macromolecules [15] and reacts with amino groups of DNA bases, the overall DNA stability is reduced [16]. Therefore, FFPE tissue samples are characterized by decreased amounts of amplifiable template DNA and sequencing artefacts [17]. Nevertheless, FFPE material is an important source for the detection of oncologically relevant variants. The method is essential when preparing tissue samples for pathological assessment and long-term storage at room temperature. Thus, optimizing upstream processes like DNA extraction, even from such challenging sample material, is important when seeking to improve overall sequencing performance [18]. Nevertheless, obtaining high coverage depths is the most relevant step in achieving high quality sequencing performance and enabling high-confidence variant detection [19,20]. 

Targeted sequencing focuses on a specific set of regions of interest with clinical relevance and, therefore, achieves higher sequencing depths and can identify variants with low frequency. This becomes especially important in the hematological malignancies that often originate from a single malignant stem cell. Here, clinically relevant variants may be already detected in pre-disease states, such as clonal hematopoiesis of indeterminate potential (CHIP) from blood or bone marrow. Importantly, CHIP is defined by the presence of a leukemia-associated mutation with a variant allele frequency (VAF) of at least 2% in the absence of a hematological malignancy [21]. Compared with hematological diagnostics, imaging techniques are—besides conventional and molecular pathology—essential for solid tumor diagnostics. Accordingly, imaging provides a method by which to non-invasively assess tumor location and size. Further, sample material is available from biopsies and surgical interventions and, although samples can be of very small size, they consist of a considerably large proportion of neoplastic cells. In contrast, in hematological malignancies, a small fraction of tumor cells often need to be detected in a large pool of healthy cells. Here, higher sequencing sensitivity and reliable detection of low allele frequencies are crucial for diagnosis, for rapid initiation of adequate therapy and, later, for monitoring. Thus, variants that may not be clinically relevant at first glance can still be used to monitor the development of malignant clones over the course of the disease. Conventional NGS allows variant detection sensitivities of 2–5%. A limit of detection (LOD) of 2% is particularly beneficial in hematological malignancies, for example, to detect aberrations with pathogenic potential that may progress over the course of a disease and for the monitoring of variants during and after treatment [22]. In solid tumor diagnostics, a LOD of 5% is adequate [23]. Deep-targeted sequencing approaches can detect VAFs as low as 0.1 or 0.2% [24,25]. This method is rarely used in diagnostics due to increased costs but is gaining interest as phenomena such as clonal hematopoiesis are discussed. However, lately targeted sequencing panels for measurable residual disease (MRD) detection are available, though they are yet to be implemented in routine MRD assessment.

The commercially available Oncomine™ Myeloid Research Assay comprises 526 amplicons representing 40 genes relevant to a wide range of tumor-associated mutations. However, not all amplicons achieve equally high coverage. This study presents a technical evaluation of the panel, including structured screening, identification and characterization of underperforming amplicons. Underperforming amplicons were compared between samples from fresh and FFPE material and were further categorized regarding their coverage of clinically relevant mutations.

## 2. Results

### 2.1. Assessment of Workflow Stability

Workflow evaluation demonstrated robust variant and VAF detection for most amplicons when comparing the results from two sequencing runs of the same sample performed in two different laboratories (for detailed analysis, see Supplementary Tables S3 and S4). Variant calls with a difference in VAF of 5% or higher between both laboratories were analyzed, and results are shown in Table 1.

When using fresh material, 31 amplicons with a differing variant call were detected, whereas 39 amplicons were identified in FFPE material input material, as shown in Table 2.

### 2.2. Underperforming Amplicons in Fresh Bone Marrow and Blood Material

Sequencing results from fresh material samples were analyzed and amplicons were classified into five performance categories based on achieved coverage across samples according to Table 6. As a result, 49 amplicons were identified as underperforming in at least 1% of all fresh material samples, as shown in Table 3 and Supplementary Table S5. Accordingly, 25 amplicons were assigned to category 0, 10 amplicons were classed into category I and 4 amplicons fulfilled the criteria of category II. Another five amplicons were found to underperform in half of all sequenced samples and were therefore classified in category III. Lastly, five amplicons underperforming in at least 76% of all fresh material samples were assigned to category IV, covering genetic regions in *ASXL1*, *BCOR*, *BRAF*, *PRPF8* and *SH2B3*.

Samples from fresh material were analyzed (n = 55), and all amplicons with coverage below 400 reads were identified as underperforming. These amplicons were sorted into five categories according to Table 6. Amplicons indicated in bold showed low performance in FFPE material, too.

Due to known problems with gene regions that are difficult to sequence, some target regions are covered by more than one amplicon in the panel. Interestingly, the majority of genetic regions in which underperforming amplicons were identified are only covered by those very amplicons. Additional amplicons covering such regions to a relevant extent are not included the panel (for detailed analysis, see Supplementary Table S7). 

For example, the amplicon BCOR_8.202477 is only covered by two base pairs by another amplicon with sufficient coverage (chrX:39923068 to chrX:39923070). The remaining uncovered part includes mutations such as p.E1190K (c.3568G>A). 

A similar lack of coverage was observed for the amplicon BCOR_6.192911, which has an overlap with another amplicon from chrX:39921447 to chrX:39921494. Amongst others, the mutation p.Q1430* (c.4288C>T) is not encompassed by this second amplicon. 

The underperforming amplicons CEBPA_1.1.16632, CEBPA_1.1.81913 and CBPA_1.1.86676 overlap to some extent, but relevant mutations were found to be located in sequence stretches covered solely by specific amplicons. For example, the mutation p.Y181* (c.543C>G) can only be assessed by amplicon CEBPA_1.1.16632, while mutation p.G223R (c.667G>C) is only covered by amplicon CEBPA_1.1.86676. 

The amplicon PRPF8_23.351225 was also identified as underperforming, but the corresponding genetic region (chr17:1577773 to chr17:1577932) was found to be covered by a second amplicon. However, the mutation p.F1099 = (c.3297C>T) is not encompassed by this overlap. 

Similarly, the region chr13:48954321 to chr13:48954356 is covered by the underperforming amplicon RB1_15.4310 as well as another adequately performing amplicon, but mutation p.R467* (c.1399C>T) is not included in this overlap. Furthermore, the amplicons RUNX1_1.29840 and SH2B3_1.100835 were identified as underperforming, resulting in insufficient coverage of mutations p.P425L (c.1274C>T) and p.A223V (c.668C>T), respectively. 

For *SH2B3*, another three amplicons were found to be underperforming. Amplicon SH2B3_1.49539 partly overlaps with another amplicon, but mutation p.R43C (c.127C>T) is not contained in this region. The underperforming amplicons SH2B3_1.52815 and SH2B3_1.85663 also partially cover each other, but exclusively covered segments include mutations such as p.I145T (c.434T>C) and p.C76Y (c.227G>A). 

Furthermore, amplicon SRSF2_1.82848 was determined as underperforming, affecting the coverage of mutation p.P95H (c.284C>A). 

In *STAG2*, a total of ten amplicons were identified as underperforming. Although underperforming amplicon STAG2_6.223906 is covered by another amplicon from chrX:123179076 to chrX:123179154, the mutation p.W168* (c.504G>A) is not encompassed by this overlap. Moreover, the underperforming amplicons STAG2_10.14939 and STAG2_10.43800 overlap to some extent, but include mutations covered by only one of them. For instance, the mutation p.Q389* (c.1165C>T) can be assessed by STAG2_10.14939, whereas mutation p.F367L (c.1101T>A) can only be sequenced with amplicon STAG2_10.43800. Additionally, mutation p.Q963* (c.2887C>T) is only covered by amplicon STAG2_26.25139. 

Amplicons TP53_1.3.1590469 and ZRSR2_11.253014 were both found to be underperforming, affecting the coverage of the mutations p.R337C (c.1009C>T) and p.R452C (c.1354C>T), respectively. 

Lastly, amplicon TP53_1.3.1810912 was identified as underperforming, covering a small part of *TP53* exon 11 and the following intron. However, the exon sequence is completely covered by another adequately performing amplicon so that the sequencing quality of this region was found to be unaffected. 

In discussions with the manufacturer, amplicons ASXL1_1.875, BRAF_18.30633 and PRPF8_12.173121 were revealed to also be rated as underperforming by them.

### 2.3. Underperforming Amplicons in FFPE Material

Sequencing runs performed with FFPE material were analyzed and underperforming amplicons were classified into the five performance categories, shown in Table 6. In total, 103 amplicons were identified as underperforming in at least 1% of samples (Supplementary Table S6). To provide a better overview, only the 21 amplicons underperforming in at least 10% of the samples are shown in Table 4. Correspondingly, ten amplicons were assigned to category I, two amplicons were sorted into category II and one amplicon was classified as category III. Lastly, 8 amplicons were found to underperform in 76 to 100% of all sequenced samples and therefore fulfilled the criteria for category IV. This category exclusively contains amplicons that were also identified as underperforming in fresh material samples (indicated in bold in Table 4) covering genetic regions in *ASXL1*, *BCOR*, *BRAF*, *CEBPA*, *PRPF8*, *RB1*, *STAG2* and *ZRSR2*.

Samples from FFPE material were analyzed (n = 59). All amplicons with coverage under 400 reads were identified as underperforming. These amplicons were sorted into five categories according to Table 6, only categories I to IV are shown. Amplicons indicated in bold showed low performance in fresh material, too.

Most of the amplicons found to be underperforming are not covered to a relevant extent by other amplicons in the panel (Supplementary Table S7). This applied to mutations such as p.E1190K (c.3568G>A), p.Y181* (c.543C>G), p.G223R (c.667G>C) or p.I145T (c.434T>C), covered by amplicons BCOR_8.202477, CEBPA_1.1.16632, CEBPA_1.1.86676 and SH2B3_1.52815, respectively. Interestingly, some amplicons were only found to underperform in samples from FFPE material. For example, amplicons BCOR_6.192911 and BCOR_12.3.360445 contain mutations that are not covered by other amplicons, such as p.S1405L (c.4214C>T) and p.P126L (c.377C>T). Similar findings were identified for amplicon SH2B3_3.75509, which includes mutations like p.T274N (c.821C>A) that cannot be assessed with other amplicons. Furthermore, the underperforming amplicon STAG2_10.14939 does not completely overlap with the adequately performing amplicon STAG2_10.43800, resulting in affected coverage for mutation p.Q389* (c.1165C>T). 

Amplicons TP53_1.3.1810912, ASXL1_1.875, BRAF_18.30633 and PRPF8_12.173121, which were already classified as low performing by the panel manufacturer, were found to also underperform in FFPE samples.

### 2.4. Comparison of Amplicon Performance in FFPE and Fresh Material

**Figure 1:**
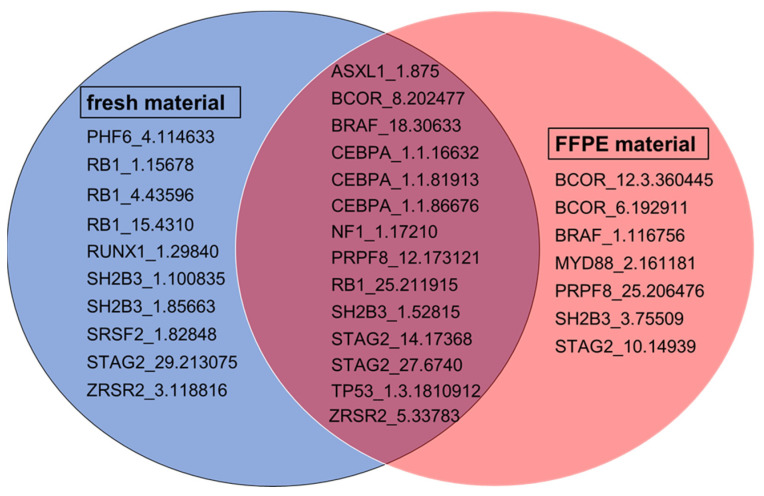
Depiction of underperforming amplicons found for fresh and FFPE material. The left circle (blue) shows underperforming amplicons detected in fresh material. The right circle (red) shows underperforming amplicons detected in FFPE material. The overlap of both circles contains underperforming amplicons detected in both materials (purple).

A comparison of amplicons underperforming in at least 10% of samples from both materials revealed a similar distribution. In samples from fresh material, 24 amplicons underperformed, whereas 21 underperformers were detected in samples from FFPE material. As shown in Figure 1, insufficient sequencing coverage was found for 14 amplicons regardless of input material.

### 2.5. Clinical Relevance of Underperforming Amplicons

In total, the analysis revealed 31 amplicons that did not achieve a coverage of 400 reads in at least 10% of all sequenced samples (see Figure 1). The clinical relevance of mutations covered by these amplicons was assigned to three different groups, as shown in Table 5. The majority of amplicons (n = 23) included mutations with clinical relevance in hematopoietic and lymphoid cancers and solid tumors. A smaller group consisted of amplicons with clinical relevance solely in solid cancer types. Only the amplicons ASXL1_1.875, BRAF_18.30633 and NF1_1.17210 cover mutations with currently unknown clinical relevance.

## 3. Discussion

NGS has been proven to be an excellent method for profiling mutation patterns in different cancer types. Thus, this method is vital in clinical diagnostics. Several studies have demonstrated the efficiency of NGS in detecting somatic alterations and exploring the mutational landscape in different cancer types using the Ion Torrent^®^ or Illumina^®^ platform [26,27,28,29,30,31]. In order to oncologically detect relevant mutations with diagnostic or prognostic significance, corresponding gene sets and respective target regions need to be carefully selected. This will help in achieving the intended applicability and reliability of the designed sequencing panel. Targeted sequencing panels are not a one-size-fits-all solution and, when seeking to detect as many variants relevant to different cancer types as possible with one panel, the coverage achieved by some amplicons will likely be insufficient. Therefore, validation and troubleshooting of complex NGS panels remains an ongoing process. This study aimed to perform a technical evaluation of the commercially available Oncomine™ Myeloid Research Assay in material from fresh and FFPE samples on the Ion Torrent^®^ NGS platform. At this point it should be noted that the Oncomine™Myeloid Panel is also fully licensed for Illumina^®^ technology, but that evaluating its performance on this platform was not within the scope of the study. 

Analyzing the respective results showed that some of the amplicons were not sequenced with the required quality in nearly all samples, independent of input material. This included amplicons covering mutation sites with varying clinical relevance in *ASXL1*, *BCOR*, *BRAF* and *PRPF8* (amplicons ASXL1_1.875, BCOR_8.202477, BRAF_18.30633, PRPF8_12.173121). Furthermore, the amplicon SH2B3_1.52815 did not reach the set quality standard in nearly all samples from fresh material. In contrast, amplicons CEBPA_1.1.81913, RB1_25.211915, STAG2_27.6740 and ZRSR2_5.33783 showed insufficient performance in nearly all samples from FFPE material, as shown in Table 3 and Table 4, category IV. The amplicons mentioned above are relevant to the assessment of mutations of clinical relevance in hematopoietic and lymphoid cancers as well as in solid tumors (see Table 5). For example, mutations in genes like *ASXL1*, *BCOR*, *STAG2* or *ZRSR2* can be used for AML classification in correspondence with the ICC guidelines from 2022 and are essential for risk-stratification and prognostic decisions in AML patients according to the European LeukemiaNet [3].

### 3.1. Assessment of Workflow Stability

To evaluate the workflow’s robustness, three samples from fresh and FFPE material were each analyzed in the laboratories in Rostock and Hannover to reveal potential differences in variant calls. Variant calls with VAFs of around 50 and 100% were chosen to evaluate technical comparability between different laboratories. These VAFs are highly likely to conform to hetero- and homozygous variants, for which corresponding expected values are known and shallow sequencing is sufficient for detecting germline alterations [20]. Both laboratories prepared sequencing libraries and performed sequencing runs according to the manufacturer’s recommendations, as described in the Materials and Methods section.

In a similar study, Haslam et al. [32] reported a high inter-laboratory concordance in VAF detection using the same sequencing platform but a different gene panel. In contrast, the presented study found VAF deviations greater than 5% between the laboratories in up to one third of all analyzed calls per sample using the Oncomine™ Myeloid Research Assay. These results possibly indicate a lack of robustness of the evaluated NGS panel. Ideally, a commercial NGS panel should compensate for changes that occur due to manual sample handling and library preparation in different laboratories to some extent. 

Further automation of the workflow helps to increase the robustness of results in a laboratory-independent manner but might also be accompanied by less flexibility when adapting process parameters with which to vary input material quality. For example, the number of PCR cycles for target amplification was increased for FFPE material in this study. Furthermore, this study only evaluated a small sample size, which may also contribute to the observed deviations. However, such circumstances are representative of small laboratories with lower sample numbers. Here, a robust workflow is essential nonetheless and such laboratories may not be able to invest in process automation. 

Another reason contributing to the observed deviations in variant detection may be incorrect base calls or alignment. High throughput NGS technologies with short reads, in particular, show error rates of up to 2% per nucleotide [20,33]. This is especially critical when NGS panels are used for in-clinic diagnostics of hematological malignancies. For this application, reliable identification of low VAF ranges between 2% and 5% is essential to detect oncologically relevant mutations in pre-disease states like CHIP, early disease states or MRD monitoring. Here, adequate sequencing sensitivity enables early diagnosis or MRD detection and subsequent initiation of suitable therapy which can significantly improve a patient’s outcome, especially in acute hematological neoplasms [4,34,35]. Therefore, consistent quality control and regular evaluation of sequencing performance are required to identify potential sources of bioinformatics errors.

### 3.2. Comparison of Amplicon Performance in Fresh and FFPE Material

When comparing amplicon performance in fresh material with that in FFPE material in this study, the latter was found to have a more inconsistent performance, with almost twice as many underperforming amplicons (Table 3 and Supplementary Table S6). This distinct difference is expected given that the sequencing performance highly depends on the quality of input material. It is known that FFPE material adversely affects NGS performance, as FFPE fixation causes DNA deamination, which then results in background noises disturbing signal measurement [10,17]. Sequencing fresh material is therefore preferred and procedures are well established, but, especially in clinical pathology, sample materials are usually prepared for long-time storage using FFPE fixation. Therefore, sequencing panels and workflows need to also be optimized for this type of input material. Optimization steps, such as the use of particular DNA polymerases, short amplicons for library preparation or pre-treatment with uracil-DNA glycosylase, may compensate for problems like formalin-induced sequencing artefacts or cytosine deamination [18,36,37]. Therefore, higher susceptibility to errors needs to be taken into consideration when analyzing FFPE samples with the Oncomine™ Myeloid Research Assay.

However, many studies have demonstrated that simple optimization steps can improve sequencing quality and that FFPE material is suitable for NGS [37,38]. The present study has confirmed this suitability and showed that the sequencing performance was similar to fresh material when comparing underperforming amplicons in at least 10% of all sequenced samples, according to categories I to IV in Table 6. A failure to meet a coverage of at least 400 reads in 10% or more of all sequenced samples may indicate a technical underperformance of the respective amplicon rather than a random failure. This selected threshold value helps to assess whether an amplicon that has achieved below-average results in a small number of sequencing runs has done so by chance or due to an ineffective technical design. For fresh input material, 24 out of 526 amplicons failed to meet the coverage requirement, whereas 21 out of 526 amplicons for FFPE material were identified as underperforming (see Table 3 and Table 4). Furthermore, a considerable overlap between amplicons underperforming regardless of input material was observed, as in Figure 1. Overall, this suggests that the performance of these particular amplicons might be rather influenced by their respective primer design and not so much by the quality of the sample material. 

Regardless of sample material, DNA quality was not further assessed in this study. Protocols implemented for routine molecular diagnostics were used, for which in-house validation showed that the extraction of DNA was adequate for sequencing. More importantly, sample collection and quality vary inherently in daily clinical routine, resulting in fluctuations in the quality of extracted DNA. As patient sample material is limited, a sequencing panel should, to a certain extent, be able to compensate for the limitations imposed by the varying quality of input material and enable reliable sequencing results from multiple sample sources. 

### 3.3. Sequencing Depth

An important strategy by which to reduce the number of underperforming amplicons is to increase the sequencing depth [19]. A thorough initial evaluation of the workflow is essential especially important when using FFPE material to assess required sequencing depths, but consistent coverage depends on many other factors. For example, large panels targeting various genetic regions and oncologically relevant variants will achieve lower sequencing coverages at the same sequencing depth than small panels targeting only specific hotspots. In addition, some cancer-relevant genes that are particularly susceptible to mutations are difficult to sequence. These may include gene segments with high sequence similarity to other regions in the genome. Sequencing reads from such regions may instead be incorrectly aligned to the non-target region and then filtered out during analysis, leading to lower coverage of the initially targeted gene segment [9]. Moreover, target regions with low or high GC content may affect PCR amplification or sequencing reaction, resulting in lower overall sequencing quality [9,39,40]. In this study, a total of 31 amplicons were found to be underperforming in at least 10% of all samples from fresh and FFPE materials. According to Aird et al. [39], optimal coverage can be expected for amplicon GC content of around 50%, whereas a GC content over 60% or lower than 30% results in substandard coverage. For the 31 underperforming amplicons identified in this study, GC content over 60% was found in 12 and GC content below 30% was determined for 3, as in Supplementary Table S8. 

An approach worth investigating might be the combination of targeted NGS with novel sequencing methods such as Nanopore sequencing by Oxford Nanopore Technologies. This sequencing technique does not rely on PCR, as DNA or RNA can be sequenced directly. Therefore, this single-molecule DNA sequencing seems to be an efficient method for sequencing GC or repeat-rich regions and appears to be a suitable addition to established NGS applications [41,42].

Overall, this study identified four amplicons as underperforming in nearly all analyzed samples, regardless of the input material (ASXL1_1.875, BCOR_8.202477, BRAF_18.30633, and PRPF8_12.173121). Here, increasing the sequencing depth may be reasonable when seeking to achieve the sequencing coverages required to reliably detect the low VAFs suitable for solid tumor diagnostics as well as hematological malignancies. However, this appears to be rather inefficient and therefore not applicable, as most of the amplicons included in the Oncomine™ Myeloid Research Assay were found to perform adequately at the described sequencing depth of one million reads per sample. Furthermore, a high GC content was determined for the underperforming amplicons ASXL1_1.875 and BRAF_18.30633, which could account for their insufficient performance. Nevertheless, no clinically relevant mutations have been deposited in the Catalogue of Somatic Mutations in Cancer (COSMIC) database for the respectively covered region at the moment of writing. As molecular diagnostics is a rapidly evolving field, clinically relevant mutations might be discovered in the following years. The other two underperforming amplicons both cover clinically relevant mutations, such as the *BCOR* mutation p.E1190K (c.3568G>A), which is relevant to MDS diagnostics and can only be assessed via the amplicon BCOR_8.202477. Similarly, the amplicon PRPF8_12.173121 enables the detection of relevant mutations, such as p.S1713G (c.5137A>G) in *PRPF8*. This mutation is observed in colorectal cancer cell lines and was found to affect mRNA processing [43]. This appears to be problematic, as clinical investigators must be able to rely on the panel to detect all relevant mutations in the targeted regions of interest. Insufficient coverage of such clinically significant loci may result in false negative diagnosis of low frequency variants in positive patient samples and lead to inadequate treatment.

## 4. Materials and Methods

### 4.1. Nucleic Acid Isolation and Quantification

In this study, 55 samples, including fresh material like blood (n = 15), bone marrow (n = 38) or lyophilized cells from molecular hemato-oncological ring trial samples (UK NEQAS, Sheffield, UK) (n = 2), were collected at the Clinic for Hematology, Oncology and Palliative Care of the Rostock University Medical Center, Germany (for detailed overview, see Supplementary Table S1). According to the manufacturer’s instructions, genomic DNA (gDNA) was isolated from all fresh material samples using the NucleoSpin Tissue kit (Macherey Nagel, Düren, Germany). Quantification was performed using the Qubit 2.0 fluorometer system and the Qubit™ dsDNA HS Assay Kit (both Invitrogen, Waltham, MA, USA).

In addition, another 59 samples from FFPE material were collected by the Institute for Pathology of Hannover Medical School, Germany. FFPE samples were prepared with the Maxwell© RSC DNA FFPE kit on a Maxwell© RSC instrument (Promega, Fitchburg, MA, USA) for gDNA isolation, and quantification was performed with a Qubit™ dsDNA HS Assay Kit on a Qubit 2.0 fluorometer system (Invitrogen, Waltham, MA, USA).

### 4.2. Targeted Sequencing

Sequencing libraries were prepared manually following the Oncomine™ MyeloidResearch Assay Manual (Thermo Fisher Scientific, Waltham, MA, USA) according to the manufacturer’s recommendations. For target amplification, 10 ng of gDNA per pool at a minimum concentration of ≥1.48 ng/μL was used. Targets were amplified using the two primer pools and a multiplex PCR running for 17 cycles for fresh material and 22 cycles for FFPE material. Calculated from primer positions, a mean amplicon length of 230 bp is to be obtained. The shortest amplicon included in the panel has a length of 80 bp and the longest amplicon has a size of 250 bp. A Taqman-based real-time PCR using the Ion Library TaqMan™ Quantitation Kit (Thermo Fisher Scientific, Waltham, MA, USA) was performed with 40 cycles for library quantification. Templating and chip loading was completed by an Ion Chef™ instrument or manually by the Ion OneTouch™ 2 system (both Thermo Fisher Scientific, Waltham, MA, USA).

Sequencing runs were performed on an Ion Torrent™ Personal Genome Machine™ system and an Ion GeneStudio S5 system (both Thermo Fisher Scientific, Waltham, MA, USA).

### 4.3. Data Analysis

The human genome assembly hg19 was used as alignment reference sequence. Samples with a minimum sequencing depth of one million reads were considered for further analysis, except for one analyzable sample with only 935,064 reads. For samples from fresh material, amplicon coverage (Coverage Analysis plugin version v5.12.0.0), variant calling (VariantCaller plugin version 5.12.0.4) and allele frequency determination were analyzed using the Torrent Suite™ software (Thermo Fisher Scientific, Waltham, MA, USA; version 5.18.1) and the Ion Reporter^TM^ (version 5.6). Analysis with the Ion Reporter^TM^ was performed with the Ion Reporter default analysis parameter settings for Oncomine Myeloid Research. Samples from FFPE materials were analyzed analogously with the same plugin and IonReporter™ versions listed above.

### 4.4. Assessment of Workflow Stability

To evaluate the robustness of the workflow, three samples from fresh material and three samples from FFPE material were each prepared in Rostock and Hannover. All calls with VAFs of approximately 50% and 100%, which most likely correspond to hetero- and homozygous variants, were analyzed per sample as respective expected values are known for such variants. Therefore, appropriate calls were chosen to assess the technical comparability of sequencing results from different laboratories. Variant calls with a difference in VAF of 5% or higher between the laboratories were defined as differing variant calls. 

### 4.5. Analysis of Underperforming Amplicons

In order to theoretically ensure a detection limit of 2% VAF, a coverage of 500 reads per amplicon is required to sequence a variant in at least ten reads per strand orientation. In this study, a coverage minimum of 400 reads per amplicon was set to identify amplicons below the quality standards. This threshold was chosen in order to provide a certain level of tolerance to technical variability occurring in real-world sequencing applications, while also enabling the reliable detection of VAFs as low as 2%. Technical variability may occur due to varying sample material quality and storage durations, different users, and fluctuations in the sequencing reaction. Amplicons failing to meet a coverage of 400 reads were further referred to as underperforming and were classified into five performance categories, as in Table 6. The categories indicate the proportion of all samples tested in which an amplicon failed to reach the coverage threshold.

The clinical relevance of mutations was defined using the COSMIC Database (cancer.sanger.ac.uk), version 92 (for detailed analysis, see Supplementary Table S2). For example, somatic mutations with a pathogenic FATHMM prediction were considered to be clinically relevant for each amplicon. Mutations found in samples from solid tumors and cancer with hematopoietic and lymphoid origin were considered.

## 5. Conclusions

In summary, this study showed that 14 amplicons included in the commercially available Oncomine™ Myeloid Research Assay did not achieve coverages sufficient for reliable variant detection in most of the analyzed samples, independent of their input material. Overall, 27 genes were identified for which sufficient sequencing performance was not achieved in fresh and FFPE material samples, including hot spot regions in *ASXL1*, *BCOR*, *BRAF* and *PRFP8*. Furthermore, comparison of used material types revealed that high quality NGS results can be obtained from FFPE samples. However, a targeted sequencing panel and its workflow must be implemented comprehensively and systematically. In addition, consistent quality control and evaluation of possible error sources are required to generate reliable sequencing data. Fully or partially automated platforms may be an option with which to increase robustness of the workflow.

## Figures and Tables

**Table 1:** Results of workflow validation by comparing detected variant allele frequency (VAF). The same samples were prepared and sequenced in both laboratories, and detected VAF were compared. Amplicons with a VAF differing for more than 5% in the same sample (differing variant calls) are listed.

Sample	Material	Number of Total Variant Calls	Number of Differing Variant Calls
1	Fresh	65	14
2	Fresh	69	20
3	Fresh	57	11
4	FFPE	61	11
5	FFPE	73	23
6	FFPE	62	14

**Table 2:** Overview of identified amplicons with differing variant calls (more than 5% difference in VAF in the same sample).

Sample Material	Amplicons with Differing Variant Calls		
Fresh material	ASXL1_13.3.346675,	ASXL1_13.3.496068,	ASXL1_6.4574,
	ASXL1_7.4911,	CBL_2.191578,	FLT3_14.216122,
	FLT3_14.74296,	IKZF1_3.61448,	MYD88_1.157213,
	NF1_13.8604,	NF1_14.128695,	NF1_26.236704,
	NF1_38.255547,	NF1_7.195821,	NPM1_1.84536,
	PRPF8_38.455070,	RB1_15.4310,	RB1_22.8039,
	RB1_25.211915,	RUNX1_3.80512,	RUNX1_9.14780,
	SF3B1_2.54904,	SF3B1_3.232163,	SF3B1_4.60700,
	SH2B3_1.100835,	SH2B3_3.75509,	STAG2_13.37196,
	STAG2_15.14340,	TET2_9.1.839229,	TP53_10.202027,
	TP53_10.97990		
FFPE material	ASXL1_13.1.451988,	BCOR_1.134332,	CBL_2.191578,
	CEBPA_1.1.16632,	EZH2_5.133393,	EZH2_15.42505,
	EZH2_17.30704,	HRAS_2.41391,	KIT_17.49555,
	MYD88_1.157213,	NF1_26.236704,	NF1_38.255547,
	NPM1_1.84536,	PRPF8_25.206476,	PRPF8_38.455070,
	RB1_3.18575,	RB1_4.43596,	RB1_22.8039,
	RB1_15.4310,	RB1_17.165046,	RUNX1_239.65068,
	RUNX1_1.136369,	RUNX1_1.29840,	SF3B1_4.60700,
	RUNX1_7.76102,	SF3B1_3.232163,	SH2B3_7.132396,
	SH2B3_1.100835,	SH2B3_3.75509,	STAG2_18.267240,
	STAG2_10.14939,	STAG2_10.43800,	TP53_9.99685,
	TP53_1.3.1810912,	TP53_8.616,	ZRSR2_5.33783
	TP53_10.202027,	WT1_2.145305,	

**Table 3:** Overview of amplicons identified as underperforming from fresh material samples. Samples from fresh material were analyzed (n = 55), and all amplicons with coverage below 400 reads were identified as underperforming. These amplicons were sorted into five categories according to Table 6. Amplicons indicated in bold showed low performance in FFPE material, too.

Category				
0	I	II	III	IV
Amplicons underperforming in percentage of all samples (n = 55)				
1–9%	10–25%	26–50%	51–75%	76–100%
Amplicons				
BCOR_6.192911	PHF6_4.114633	RB1_4.43596	**CEBPA_1.1.16632**	**ASXL1_1.875**
BCOR_10.16915	RB1_1.15678	**RB1_25.211915**	**CEBPA_1.1.81913**	**BCOR_8.202477**
JAK2_4.68202	RB1_15.4310	RUNX1_1.29840	**CEBPA_1.1.86676**	**BRAF_18.30633**
NF1_55.95970	SH2B3_1.100835	**TP53_1.3.1810912**	**NF1_1.17210**	**PRPF8_12.173121**
NPM1_1.84536	SRSF2_1.82848		SH2B3_1.85663	**SH2B3_1.52815**
PHF6_2.86405	**STAG2_14.17368**			
PHF6_6.45032	**STAG2_27.6740**			
PRPF8_23.351225	STAG2_29.213075			
PRPF8_25.206476	ZRSR2_3.118816			
PRPF8_42.75713	**ZRSR2_5.33783**			
RB1_6.27164				
RB1_9.72315				
SF3B1_5.8195				
SH2B3_1.49539				
STAG2_6.223906				
STAG2_9.168558				
STAG2_10.14939				
STAG2_10.43800				
STAG2_15.14340				
STAG2_26.25139				
STAG2_33.62661				
TET2_3.23136				
TP53_1.3.1590469				
ZRSR2_7.7640				
ZRSR2_11.253014				

**Table 4:** Overview of amplicons identified as underperforming from formalin-fixed paraffin-embedded (FFPE) material samples. Samples from FFPE material were analyzed (n = 59). All amplicons with coverage under 400 reads were identified as underperforming. These amplicons were sorted into five categories according to Table 6, only categories I to IV are shown. Amplicons indicated in bold showed low performance in fresh material, too.

Category			
I	II	III	IV
Amplicons underperforming in percentage of all samples (n = 59)			
10–25%	26–50%	51–75%	76–100%
Amplicons			
BCOR_6.192911	BCOR_12.3.360445	PRPF8_25.206476	**ASXL1_1.875**
BRAF_1.116756	**CEBPA_1.1.86676**		**BCOR_8.202477**
**CEBPA_1.1.16632**			**BRAF_18.30633**
MYD88_2.161181			**CEBPA_1.1.81913**
**NF1_1.17210**			**PRPF8_12.173121**
**SH2B3_1.52815**			**RB1_25.211915**
SH2B3_3.75509			**STAG2_27.6740**
STAG2_10.14939			**ZRSR2_5.33783**
**STAG2_14.17368**			
**TP53_1.3.1810912**			

**Table 5:** Overview of underperforming amplicons grouped by clinical relevance of covered mutation sites. Shown are all amplicons detected as underperforming in fresh material and FFPE material (n = 31) in at least 10% of the samples. Amplicons were sorted into three groups based on the clinical relevance of mutations detected by them.

Clinical Relevance.	Clinically Relevant in Cancer Diseases with Hematopoietic/Lymphoid Origin and Solid Cancer Types [n = 23]	Clinically Relevant Solely in Solid Cancer Types [n = 5]	No Clinical Relevance Known to Date [n = 3]
Amplicons	BCOR_6.192911	BRAF_1.116756	ASXL1_1.875
	BCOR_8.202477	PRPF8_12.173121	BRAF_18.30633
	BCOR_12.3.360445	PRPF8_25.206476	NF1_1.17210
	CEBPA_1.1.16632	RB1_4.43596	
	CEBPA_1.1.81913	TP53_1.3.1810912	
	CEBPA_1.1.86676		
	MYD88_2.161181		
	PHF6_4.114633		
	RB1_1.15678		
	RB1_15.4310		
	RB1_25.211915		
	RUNX1_1.29840		
	SH2B3_1.100835		
	SH2B3_1.52815		
	SH2B3_1.85663		
	SH2B3_3.75509		
	SRSF2_1.82848		
	STAG2_10.14939		
	STAG2_14.17368		
	STAG2_27.6740		
	STAG2_29.213075		
	ZRSR2_3.118816		
	ZRSR2_5.33783		

**Table 6:** Categories Categories of underperforming amplicons based on their underperforming proportion in all investigated samples.

Performance Categories	Amplicons Underperforming in Percentage of All Samples
Category 0	1 to 9%
Category I	10 to 25%
Category II	26 to 50%
Category III	51 to 75%
Category IV	76 to 100%

## Data Availability

The raw data supporting the conclusions of this article will be made available by the corresponding author upon reasonable request.

## Supplementary Materials

The supporting information can be downloaded at: https://www.mdpi.com/article/10.3390/ijms25063534/s1.
